# Posterior cortical atrophy: correlation between clinical, neuropsychological, magnetic resonance and positron emission tomography Imaging

**DOI:** 10.1186/1750-1326-8-S1-P11

**Published:** 2013-09-13

**Authors:** Tinatin Chabrashvili, Haley Lamonica, Bassel Sawaya

**Affiliations:** 1 Tufts Medical Center, Tufts University School of Medicine, Boston, MA, USA; 2 Temple University School of Medicine, Philadelphia, PA, USA

## Background

Posterior cortical atrophy is a rare syndrome and considered to be a spectrum of Alzheimer’s disease. Cases of corticobasal degeneration, dementia with Lewy Body, and prion diseases, such as Creutzfeldt-Jakob disease, have also been described with such a syndrome. Despite the similar pathological findings seen in Alzheimer’s disease, patients with posterior cortical atrophy present with prominent visuoperceptual impairment which is disproportional to impairment in other cognitive domains. Frequently, these patients are initially seen in ophthalmology clinics due to their visual complaints, and often the diagnosis is delayed. Recently, Positron Emission Tomography based on the identification of amyloid burden in the brain gained momentum as a diagnostic imaging option in patients with Alzheimer’s disease. Here we present a case of a 79 year old woman with a visual disturbance, and contrast her clinical presentation with neuropsychological testing and Magnetic Resonance and Positron Emission Tomography imaging to demonstrate the utility of diagnostic tools in patients with a presumed diagnosis of posterior cortical atrophy.

## Materials and methods

79 year old right-handed woman with a two-year history of a “visual disturbance”, described as blurred vision, stating that during reading the letters start to merge which makes her reading challenging. She reports difficulty writing due to unknowingly inverting the letters, difficulty expressing her thoughts in writing, difficulty understanding written sentences and finding words, and more recently, mild memory problems concerning day-to-day activities.

## Results

Neurological examination was unremarkable except for higher-order visual disturbances such as optic ataxia and cognitive examination. Neuropsychological testing showed a prominent visuospatial and perceptual deficit, in conjunction with constructional apraxia, optic ataxia, alexia and anomia. Magnetic resonance imaging demonstrated a prominent cortical atrophy and mild chronic small vessel disease. Positron Emission Tomography imaging with Florbetapir-F18 showed diffuse increased radiotracer uptake in the gray matter involving occipital lobes bilaterally, right greater than left, as well as the temporal lobes. Also, a mild diffuse increased radiotracer uptake in the gray matter of the parietal lobes bilaterally was identified.

## Conclusions

The clinical presentation remains the most important aspect in the diagnosis of posterior cortical atrophy. Neuropsychological testing can aid in differential diagnosis. While the magnetic resonance imaging of brain is essential to rule out a brain structural lesion, Positron Emission Tomography imaging could be more sensitive to identify the anatomical areas with more significant amyloid deposition. Although Positron Emission Tomography imaging with Florbetapir-F18 cannot be used solely for the diagnosis of Alzheimer’s disease and related disorders, it may be helpful in clarifying the diagnosis of atypical forms such as a posterior cortical atrophy, which may be especially helpful in the future to select the appropriate patients once better treatment options are available.

